# Optimal path-finding through mental exploration based on neural energy field gradients

**DOI:** 10.1007/s11571-016-9412-2

**Published:** 2016-09-30

**Authors:** Yihong Wang, Rubin Wang, Yating Zhu

**Affiliations:** East China University of Science and Technology, Shanghai, 200237 China

**Keywords:** Cognitive map, Mental exploration, Energy coding, Energy field, Energy field gradient

## Abstract

Rodent animal can accomplish self-locating and path-finding task by forming a cognitive map in the hippocampus representing the environment. In the classical model of the cognitive map, the system (artificial animal) needs large amounts of physical exploration to study spatial environment to solve path-finding problems, which costs too much time and energy. Although Hopfield’s mental exploration model makes up for the deficiency mentioned above, the path is still not efficient enough. Moreover, his model mainly focused on the artificial neural network, and clear physiological meanings has not been addressed. In this work, based on the concept of mental exploration, neural energy coding theory has been applied to the novel calculation model to solve the path-finding problem. Energy field is constructed on the basis of the firing power of place cell clusters, and the energy field gradient can be used in mental exploration to solve path-finding problems. The study shows that the new mental exploration model can efficiently find the optimal path, and present the learning process with biophysical meaning as well. We also analyzed the parameters of the model which affect the path efficiency. This new idea verifies the importance of place cell and synapse in spatial memory and proves that energy coding is effective to study cognitive activities. This may provide the theoretical basis for the neural dynamics mechanism of spatial memory.

## Introduction

The concept of the cognitive map proposed by Tolman can be used to solve the navigation problems in environment such as self-locating, target-searching and path-finding (Tolman 1948), which caught wide attention in the field of neuroscience. He proposed that a cognitive map can be formed in the brain to represente the change in surrounding environment duiring the rat moving in the environment (Tolman 1948), which can be used to solve the spatial location problem. Place cells in hippocampus are the biological foundation of cognitive map, and they were first found by O’Keefe and Nadel (1978) in the hippocampus with an electrophysiological method. Redish and Touretzky (1997) found that the hippocampus possesses ability of spatial memory associated with spatial navigation in rodent animal. There are two kinds of neural network models in the theoretical study of the cognitive map. First is spatial vector map, representing self-location. Second is goal-oriented vector map, indicating the position of the target (Zhu et al. 2013). In the model of spatial vector map, the role of the map is to express the location of the agent in space, and the agent can be located on the map. Wilson and McNaughton (1993) recorded the firing pattern of place cell during the process of spatial exploration in animal experiments. And Wilson’s research revealed how the animal’s position in a two-dimensional plane can be expressed by place cells, which verified the efficiency of population coding. This theory showed that the position-coding with concentrated firing is linked through the interaction between closely connected neurons (Redish 1999). Some other experimental studies further verified that population coding could be restructured according to the changes of environment (Muller and Kubie 1987; Bostock et al. 1991; Gothard et al. 1996). Based on these researches, Muller, and his colleagues proposed that the place cells firing together could form a network, which made place cells to interact forming a tightly connected synapse (Muller et al. 1991; Muller et al. 1996). The main function of cognitive map model concerning the goal-oriented vector map is to navigate between the different spatial locations. Burgess et al. (1994), Blum and Abbott (1996), Gerstner and Abbott (1997), Redish and Touretzky (1998) and Trullier and Meyer (2000) made thorough researches in this kind of model. The core of their model was the asymmetric plasticity connection of pyramidal cells hippocampus, and then Hebbian plasticity learning algorithm was adapted to the model so as to generate asymmetric connection. The deficiency of this model was that it was difficult to generate asymmetric connection in spatial tasks with multi-targets. Later, Redish et al. combined the two cognitive maps in 1998 to make symmetric and asymmetric connections coexist. There have been many promising works about neural coding and the cognitive map in the recent years (Han et al. 2014; Duch and Dobosz 2011; Strauss et al. 2010; Sato and Yamaguchi 2009; Huyck 2009; Wagatsuma and Yamaguchi 2007). And there are also many wonderful pioneering researches on neural dynamics model which set up a framework for the theory and application of neural system model (Adeli and Park 1998; Park and Adeli 1995, 1997; Adeli and Karim 1997; Senouci and Adeli 2001; Adeli and Kim 2001; Tashakori and Adeli 2002; Ahmadkhanlou and Adeli 2005). However, the deficiency was that it took a large number of physical explorations to form path vector. This means to explore spatial environment continually through the actual movements, which consume time and energy. Our study can make up the defects, and physical exploration can be improved to mental exploration.

Mental Exploration was firstly proposed by Hopfield (2010). He adapted plane attractor to the energy function and substituted the mental exploration in the virtual space for the heavy process of physical exploration. The main learning algorithm was to calculate the increase of connection strength among synapses. The accumulation process began at the moment a mental position matches a physical location and terminates at the moment when special neurons send the signal “find the target”. Mental exploration has some obvious advantages compared to physical exploration. However, the study proposed by Hopfield was carried out in the artificial neural network, without direct physiological significance. Furthermore, during the process of finding an efficient path to mental exploration, there is no demand for learning speed and path efficiency. Other researchers combine spatial vector and goal-orientation maps to form a path finding mechanism (Zhu et al. 2013). And this mechanism is applied to mental exploration. But the efficiency is also not ideal. It takes at least 15 times to generate a relatively efficient path and the model is easy to be trapped into a local zone and never gets out. Furthermore, energy coding method has been gradually established and studied based on structural neural network (Wang and Zhang 2006, 2007; Wang et al. 2008, 2009), but how to apply this new method into functional neural network remains to be discussed. In this work, based on Hopfield theory, neural energy coding theory with clear biological significance is adopted (Wang and Zhang 2006, 2007; Wang et al. 2008, 2009), and the firing power of place cell is regarded as the breakthrough point to guide mental exploration. An efficient mental exploration path can be achieved with this method, which possesses the function of path optimization.

Path finding and optimization are essentially problems of neural information coding, which is widely studied and rapidly developed in the cognitive and computational neuroscience fields (Sato and Yamaguchi 2009; Wang and Zhang 2006; Wang et al. 2015; Mohemmed et al. 2012; Luque et al. 2012; Rossello et al. 2012; Ramanathan et al. 2012; Yamanishi et al. 2012; Fukushima et al. 2007). In particular, energy coding has drawn increasing attention of researchers. This coding method is adopted to guide mental exploration in our study. Energy coding of the cognitive process was developed based on the studies by Wang and Zhang (2006, 2007) and Wang et al. (2008, 2009). The core argument of these studies is that neural information can be expressed by neural energy. Thus, neural processing can be contained in the theoretical framework of global neural coding in the brain (Wang et al. 2015a, b; Wang and Wang 2014). The action potential and energy consumption in the action potential firing process has exclusive corresponding relation. It is possible for the neural information processing to perform research using energy coding (Wang et al. 2015).Neural network is a high-dimensional nonlinear complex dynamic system composed of large amounts of neurons, and neural energy can be superimposed, which provides convenience for modeling and computational analysis to reduce the cost of study. Nervous energy may be an effective tool to study the global behavior of brain activities (Wang et al. 2015; Zheng et al. 2012). In this work, based on the advantages of energy coding such as simplicity and globality, mental exploration is guided by the global neural energy field generated by the firing power of place cells. Based on these researches, this study discusses the specific problem of path-finding: a rat can initiate a random search in a strange environment, and will finally locate the target. With the increase of the number of the experiment, the rat can find the target faster, and the efficiency will be improved. Because place cell would fire according to a certain order during the experimental process, which changes the synaptic connection, the location, spatial environment and target information of a rat can be decoded by the firing pattern of group activities of place cells. The efficiency of path finding can be improved by integrating the information in the hippocampus. Based on the theoretical framework of energy coding, spatial memory is simulated in this study by constructing a new computing model and routing process. Moreover, mental exploration proposed by Hopfield is adopted (Hopfield 2010) and physical exploration is improved by mental exploration, which is endowed with clearer biological significance, so that a new navigation model with mental exploration is generated.

**Fig. 1:**
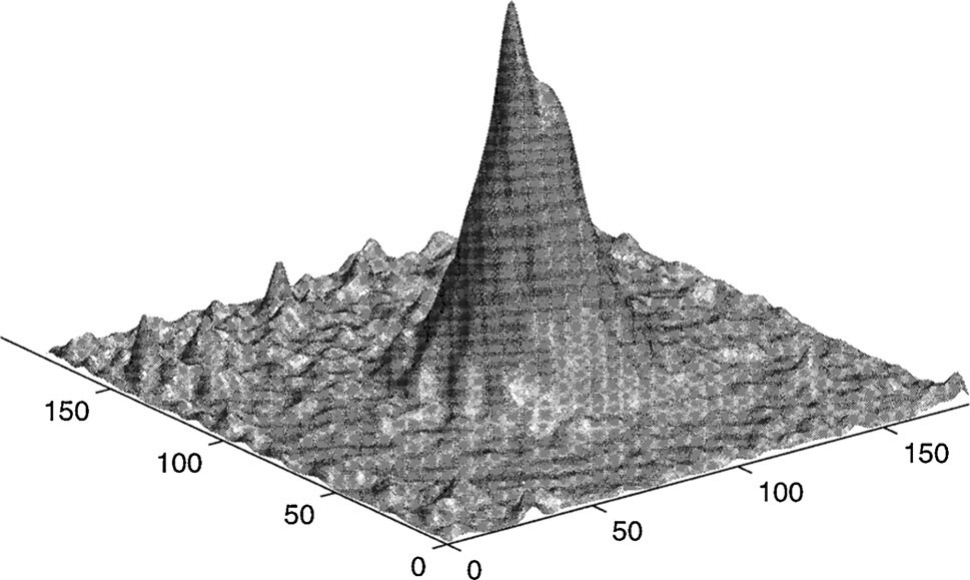
Place cells activity packet on a chart constructed from the experimental data

The firing rule of place cell responding to spatial location is as follows: when a rat is in the center of place field represented by the place cell, this place cell will lead to heavy activity and the firing rate will also be at the highest. When a rat is away from this center, the activity intensity of this place cell and its firing rate will both decrease. Figure 1 is a typical momentary distribution of the firing rate over a chart (in allocentric coordinates). The animal is located at the center of the square and is moving to the left and toward the viewer (Samsonovich and McNaughton 1997). A fixed Gaussian shape of the activity packet is introduced by Samsonovich and McNaughton (1997). The spike on top is an effect of the animal moving.

The waveforms of action potential are identical, so the number of spikes can measure the energy consumption of place cell. The number of spike number in the unit of time represents firing rate, reflecting the firing power of place cell. Based on the physiological fact mentioned above, it is possible to assume in this paper that the energy consumed by the isolated specific place cell (firing power) meets the two-dimensional Gaussian distribution. That is to say, when the system is located in the center of place field of a place cell, the firing power of this place cell will be at the highest. When the system is away from this center, this place cell declines in accordance with squared exponential of distance, and the coordinate of the place field center is regarded as expectation, which meets the two-dimensional Gaussian distribution. ‘Isolated’ means that without the influence of synaptic connections from other place cell, the place cell studied meets the firing law.

The activity of a place cell in the hippocampus corresponds to the spatial location of the animal, and place cells with different place field can spike with time order according to the movement of the animal. In accordance with the rule of ‘connect after simultaneous spike’ (Wang et al. 2015), such spikes lead to strengthening of the synaptic between place cells. Then, such strengthening effect will affect the spike of the subsequent place cell, so that every place cell can be decoded by phase-shift coding (Zhu et al. 2013). When a system moves from the place field of one position to the place field of another, this forward channel will be strengthened; that is to say that the path has been learned. When the animal is located on the path experienced before, the activated (spike with high energy) place cells can activate the place cells in their forward path through strengthening synaptic. When a rat explores maze, place cell firing results in a weighted matrix in the CA3 region of hippocampus, resulting in distinct place fields according to Hebbian long-term potentials (LTPs). LTP is a persistent strengthening of synapses based on recent patterns of activity. These are patterns of synaptic activity that produce a long-lasting increase in signal transmission between neurons. As a result, the strength of connections between place cells with overlapping areas is strengthened considerably, distinguishing these from non-overlapping areas. Then the shift vector generated will point to the front of the path. If enough paths with LTP are generated, these shift vectors can reflect the history of space exploration and a helpful map for further navigation is provided (Blum and Abbott 1996). In our model, we generate the synaptic in a way such that if two place cells spike with high power one after another, the synaptic connection between them will be strengthened once, similarly to the previous study. Meanwhile, exploration and mental exploration of an animal are always based on the target navigation, so the place cell expressing the target and the present place cell would spike with high power simultaneously, which leads to the synaptic connection between the place cell and the target cell.

## Model and method

Assuming a one-on-one correspondence between the place field center of a place cell and the node of spatial map after reticulation, a place cell and its place field center from reticulation map can represents each other when a proper reversible linear transformation is adopted. So we can use place cells to represent actural space to construct a vitural plane. Assuming there are n locations and n place cells are representing them but not in the convienient order. For example, location L_i_ and L_i+1_ are neighbours while place cells p_i_ and p_i+1_ which represent them are not. Then we can apply an certain n × n permutation matrix (transformation) to the place cell vector to move p_i_ and p_i+1_ near each other. These transformations will consist a permutation group of order n. This adoption helps to generate a virtual map to express spatial location more easily with place cell clusters. When a place cell fire independently, what is the distribution property of the firing power? Based on the consideration of physiological facts (Samsonovich and McNaughton 1997) and mathematical convenience, there are two basic hypotheses for the deduce of power distribution: (1) Activity intensity of a place cell with respect for x axis is independent from that for y axis, vice versa. In other words, the firing power of a place cell in two orthogonal direction is independent from each other. (2) Firing power distribution has rotational symmetry in space, that is to say, firing power is only dependent on the distance from center. We take the center of place field as origin of coordinates. According to hypotheses (1), power distribution P_0_(x, y) is as the following form:

1 $$P_{0} (x,y) = f(x) \times f(y)$$

Transformed into polar coordinate:

2 $$P_{0} (x,y) = P_{0} (r\cos \theta ,r\sin \theta ) = g(r,\theta )$$

According to hypotheses (2), P_0_(x, y) is only the function of distance, then we have

3 $$f(x) \times f(y) = P_{0} (x,y) = g(r) = g\left( {\sqrt {x^{2} + y^{2} } } \right)$$

Take y = 0, we can get $f(x) \times f(0) = g(x)$. Similarly, $f(y) \times f(0) = g(y)$. So

4 $$\ln \frac{f(x)}{f(0)} + \ln \frac{f(y)}{f(0)} = \ln \frac{{g\left( {\sqrt {x^{2} + y^{2} } } \right)}}{{f^{2} (0)}} = \ln \frac{{f\left( {\sqrt {x^{2} + y^{2} } } \right)}}{f(0)}$$

Replace $\ln \frac{f(x)}{f(0)}$ by h(x), we will get

5 $$h(x) = \ln \frac{f(x)}{f(0)}$$

6 $$h(x) + h(y) = h\left( {\sqrt {x^{2} + y^{2} } } \right)$$

If the function is smooth (or differentiable), this equation can be solved. For any integer n, we can get

7 $$n^{2} \cdot h(x) = h\left( {\sqrt {n^{2} \cdot x^{2} } } \right) = h(n \cdot x)\;,\;{\text{so}}\;h(n \cdot x) = n^{2} \cdot h(x)$$

Similarly,

8 $$h(x) = h\left( {m \cdot \frac{x}{m}} \right) = m^{2} \cdot h\left( {\frac{x}{m}} \right)\;,{\text{so}}\;h\left( {\frac{x}{m}} \right) = \frac{1}{{m^{2} }}h(x)$$

Then,

9 $$h\left( {\frac{n}{m}x} \right) = \frac{{n^{2} }}{{m^{2} }}h(x)$$

This means for any rational number q, we can get $h(q \cdot x) = q^{2} \cdot h(x)$. Since all rational numbers Q is a density subset of all real numbers R and the function is continuous, for any real number r, we will get

10 $$h(r \cdot x) = r^{2} \cdot h(x)\;,\;{\text{so}}\;h(x) = h(x \cdot 1) = h(1) \cdot x^{2}$$

and,

11 $$f(x) = f(0) \cdot \exp \left\{ {h(1) \cdot x^{2} } \right\}\;,\;P_{0} (x,y) = f(x) \times f(y) = a \cdot \exp \left\{ {b \cdot \left( {x^{2} + y^{2} } \right)} \right\}$$

In these equations,$a = f^{2} (0),b = h(1)$ are constant. On the condition of normalization,

12 $$\iint\limits_{R \times R} {P_{0} (x,y)dxdy} = 1$$

We can get $a = - \frac{b}{\pi },b < 0$, take $b = - \frac{1}{{2\sigma^{2} }}$, then

13 $$P_{0} (x,y) = \frac{1}{{2\pi \sigma^{2} }} \cdot \exp \left\{ { - \frac{{x^{2} + y^{2} }}{{2\sigma^{2} }}} \right\},$$

Finally, we will have the following generalization form,

14 $$P_{0} = \frac{1}{{2\pi \sigma_{1} \sigma_{2} }}\exp \left\{ { - \frac{1}{2}\left[ {\frac{{\left( {x - \mu_{1} } \right)^{2} }}{{\sigma_{1}^{2} }} + \frac{{\left( {y - \mu_{2} } \right)^{2} }}{{\sigma_{2}^{2} }}} \right]} \right\}$$

When a single place cell is fired independently, the firing power shows two-dimensional Gaussian distribution with respect to space map. When the place cell is fired due to the stimulation from excitatory synaptic of another place cells, the power obtains a certain proportion of increase, and this proportion depends on the strength of the synaptic connection. Since all the place cells possess identical action potential and similar structure and function, their firing power distributions are identical (with the same peak, variance and shape, so that they can completely overlap after translation) except the expectations which represent place field centers.

**Fig. 2:**
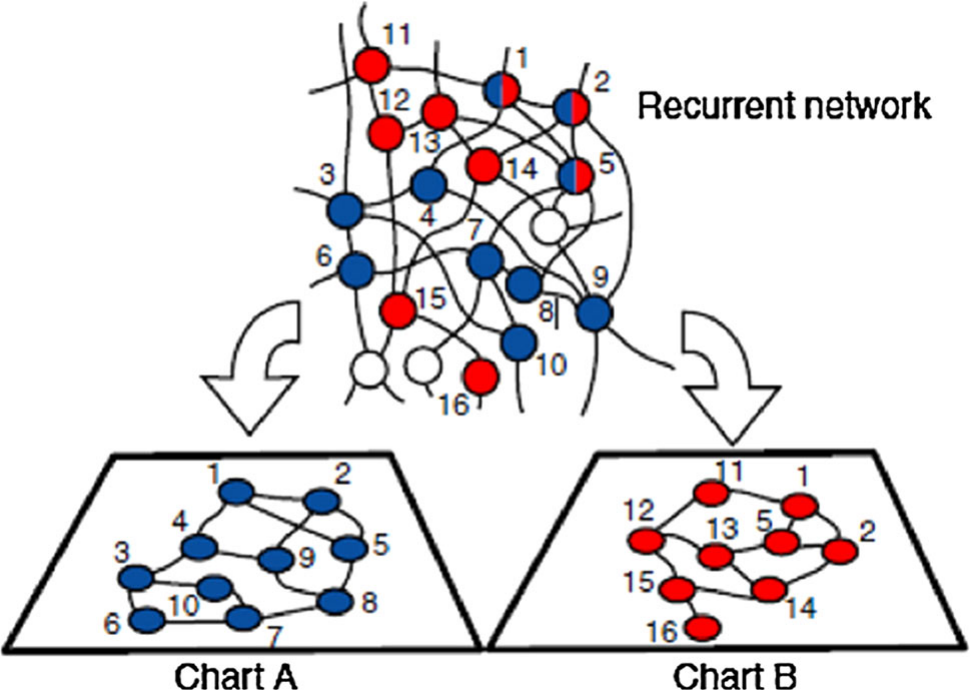
Network structure

After normalization with the maximum firing power, as we deduced before, the space distribution relative to the isolated firing power of place cell is described as Eq. (14). Where P_0_ is the normalized activity power of a particular place cell with respect to a certain location before learning. (μ_1_, μ_2_) represents the coordinate of the place field center of a place cell, and (x, y) represents the present location of the system. $\sigma_{1} ,\sigma_{2}$ define the decay rate of firing power when system is away from place field center, which can be determined by experimental data. When system is at (x, y) without the influence of another place cell synaptic, the normalization firing power of the place cell whose place field center is located at (μ_1_, μ_2_) can be calculated from the equation. The power model is continuous for the spatial location (x, y). Figure 2 is a schematic illustration of a multiple chart network (Wagatsuma and Yamaguchi 2007). The recurrent network of place cells has anatomically twisted synaptic connections. Blue and red circles, respectively, denote place cells that are active in environment A, called chart A, and the cells that are active in environment B, called chart B. The top figure represents the anatomical position of cells, and the bottom figures represent imaginary arrangements of cells that are aligned according to their place fields in environment A (blue) and environment B(red). Twisted connections in the top figure can be straightened in the bottom figures as neighboring connections among cells. Different colors means there are different cognitive maps of each environment. When construct the model, virtual plane which represents environment should consists of place cells, so a linear transformation is adopted and the place cell network structure is rearranged as a lattice. In other words, every place cell is connected to its four neighbors. There are very weak symmetric connections before exploration. This is the initial status of the network. Asymmetric connections are added to the network during exploration.

If there exists excitatory synaptic connection from another place cell to the ith cell, then the firing power will obtain a certain proportion of increase based on the original, and this ratio is determined by the strength of synaptic connection. Normalized power is given by

15 $$P_{c} = \left( {1 + \omega_{ti} + \sum\limits_{j \ne i}^{n} {\omega_{ji} } } \right)\frac{1}{{2\pi \sigma_{1} \sigma_{2} }}\exp \left\{ { - \frac{1}{2}\left[ {\frac{{\left( {x - \mu_{1} } \right)^{2} }}{{\sigma_{1}^{2} }} + \frac{{\left( {y - \mu_{2} } \right)^{2} }}{{\sigma_{2}^{2} }}} \right]} \right\}$$

16 $$k\frac{{d\omega_{ji} }}{dt} = \int\limits_{0}^{\infty } {\left[ {P_{i} (\tau )H(\tau )P_{j} (t - \tau ) + P_{i} (t - \tau )H( - \tau )P_{j} (\tau )} \right]} d\tau$$

where P_c_ is the normalized power with respect to a certain location after learning. ω_ti_ represents the synaptic connection from the object to the ith place cell, and ω_ji_ stands for the synaptic connection from other place cells to the ith one. As described in Eq. (16), Hebbian learning rule is adopted to calculate the synaptic connection strength, but this calculation is in an energy form. Because P_i_ (t) and P_j_ (t) are the power of place cell i and j at moment t. k is a constant and H (t) is a time window. Assume that the power is enhanced in a certain proportion so that all synaptic strength can be summed as a total proportion of power increase. The meanings of other factors in the Eq. (15) are consistent with Eq. (14). When rodent animal is at the position (x, y) on the plane, coordinate z is the representation of the normalized power of this particular place cell, and every place cell has a similar power distribution. At a particular moment, the animal’s location is determined, so the firing power of every place cell is determined, too. The centers of place fields are taken as independent variables, the power of corresponding place cells as z(function of (x, y)), so that the power of place cell population becomes a field defined by centers of place fields. Since the neural energy field introduced here is only defined on centers of place fields, it is a discrete field distinguished from continuous mathematical field. With the absence of synaptic connection, it is obvious that this field also acquires a Gaussian distribution form. This is the initial status of the place field without synaptic connection, which exhibits unimodal Gaussian distribution before learning, however it will become a multi-modal place fields with two or more peaks in the explored environment after learning. More details will be presented in the ‘Results’ part.

The spatial memory and the information of paths explored by an animal are stored as synaptic connections between place cells. Changing of the synapses alters the distribution of the energy field. Notably, two types of synapses appear in our model. One type of synapse is generated between adjacent place cells due to their successive high power firing. The other is generated because place cells represent current location and the target fire simultaneously with high power. Since the target location is fixed, the influence on place cells from the second type of synapse could be performed by an energy field whose center is the target location. Finally, the distribution form of the energy field on a virtual plane is determined by the following equations:

17 $$\begin{aligned} P_{k} (x,y) = P_{k0} (x,y) + P_{kt} (x,y); \hfill \\ P_{k0} (x,y) = \left( {1 + \sum\limits_{j \ne i}^{n} {\omega_{ji} } } \right)\frac{1}{{2\pi \sigma_{1} \sigma_{2} }}\exp \left\{ { - \frac{1}{2}\left[ {\frac{{\left( {x - x_{p} } \right)^{2} }}{{\sigma_{1}^{2} }} + \frac{{\left( {y - y_{p} } \right)^{2} }}{{\sigma_{2}^{2} }}} \right]} \right\}; \hfill \\ P_{kt} (x,y) = \frac{1}{{2\pi \sigma_{1} \sigma_{2} }}\exp \left\{ { - \frac{1}{2}\left[ {\frac{{\left( {x - x_{t} } \right)^{2} }}{{\sigma_{1}^{2} }} + \frac{{\left( {y - y_{t} } \right)^{2} }}{{\sigma_{2}^{2} }}} \right]} \right\} \hfill \\ (k = 1,2,3{ \ldots }) \hfill \\ \end{aligned}$$

P_k_ means the energy field after the system performs the kth step. P_k0_ is the energy field generated by place cell population. Meanwhile, synapses connecting ordinary place cells and target place cell increase the firing power, and this effect is expressed by P_kt_, which will be summed with the original energy field of P_k0_. (x, y) denote any place field centers on the plane. Moreover, (x_p_, y_p_) and (x_t_, y_t_) show the current location of the system and that of the target respectively. The power at (x, y) can be calculated with these equations when spatial position (x, y) is given. (x, y) can only be taken to be center coordinates of place field because of the discreteness of the energy field. To summarize, place cells represent spatial locations, and have firing power themselves, so these firing power could be considered as a field distributed in space. The distribution form of this field is influenced by synaptic connections among the place cells. The gradient of this energy field can be used to guide the mental exploration on virtual plane which is also represented by place cells. Then the path-finding and path-optimizing task would be accomplished by altering field gradient through changing synaptic connections. When the system finishes kth step at a certain position, the direction of next step will be determined by the direction vector $\vec{n}_{k + 1}$.

18 $$\begin{aligned} \vec{n}_{k + 1} = \frac{{\vec{\tilde{n}}_{k + 1} + \vec{n}_{rand} }}{{\left| {\vec{\tilde{n}}_{k + 1} + \vec{n}_{rand} } \right|}} \hfill \\ \vec{\tilde{n}}_{k + 1} = gradP_{k} \left( {x_{k0} ,y_{k0} } \right) \hfill \\ = \left( {\frac{{\partial P_{k} }}{\partial x}\vec{i} + \frac{{\partial P_{k} }}{\partial y}\vec{j}} \right)\left| {_{{(x,y) = \left( {x_{k0} ,y_{k0} } \right)}} } \right. \hfill \\ \end{aligned}$$

In this equation, $\vec{n}_{k + 1}$ is the normalized direction vector of next step, whose main part is $\vec{\tilde{n}}_{k + 1}$, which is determined by current energy field gradient. Gradient is calculated with discrete method because the energy field is discrete. $\vec{n}_{rand}$ is a random vector to simulate disturbance, whose direction changes randomly. The direction of $\vec{n}_{rand}$ is uniformly distributed from 0 to 2π. A particular neuron will be needed to end mental exploration by firing spikes after target is found (Hopfield 2010). In this model, exploration will be terminated when the distance between target position and real-time position of exploration is shorter than a certain distance, which is called crucial distance.

According to this model, we use Matlab (R2013a) for simulation. The path-finding process is performed in a square region of space. 225 (15 × 15) place cells are set during simulation. Target location is set to (10, 10), and system (model rat) enter this unexplored space through (1, 1) to execute the path-finding task with step length of 1 (Zhu et al. 2013). Meanwhile, crucial distance is set to be the length of 2 steps.

## Results

The path-finding process has two stages. The first one is learning in which the system (artificial animal) initiate a random search in the new environment. The goal is to get the whole layout of the environment, and the position of the target, then construct the place cells into a complete virtual plane. The second one is path-finding based on the energy field gradient. In this stage, the system guided step by step by the energy field gradient conduct mental exploration in the virtual plane and try to find an efficient path in a shortest possible time to navigate for the later physical exploration.

**Fig. 3:**
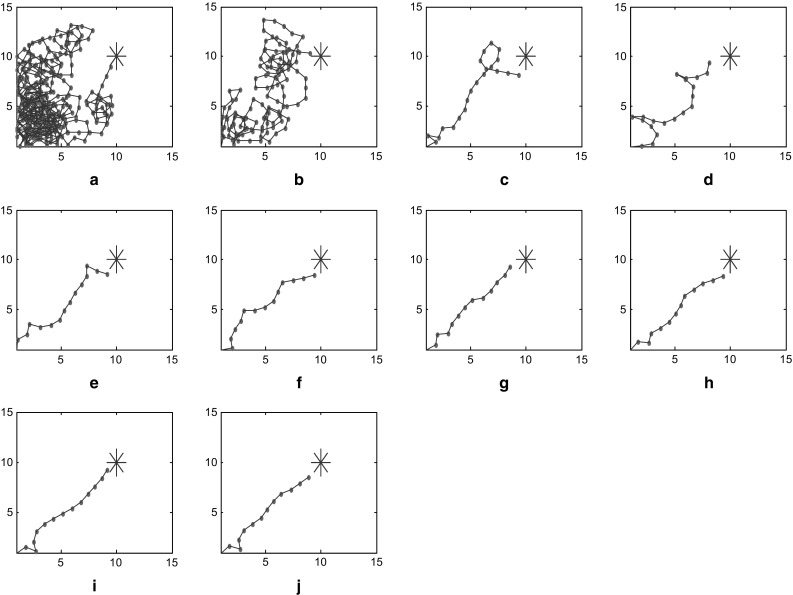
Trajectories of path finding process

Ten times of continuous operation results are shown in Fig. 3 It displayed the trajectories in virtual plane during the path-finding process. The larger blue star represents the target position to be explored, and the smaller red star represent the position of each step. The blue thread connecting them shows the motion trajectory. Figure 3a shows the first path-finding. In this case, target is unknown, and energy field is unconstructed, so the navigation vector is determined only by the random part. This is called completely random path-finding. Completely random path-finding will cover almost the whole environment, and the shorter the crucial distance is set, the more likely this path-finding will cover a larger space. However, the number of steps will increase as well. Once the first path-finding locates the target, the corresponding place cell representing this point will be activated. The later mental exploration will be navigated by energy field gradient.

**Fig. 4:**
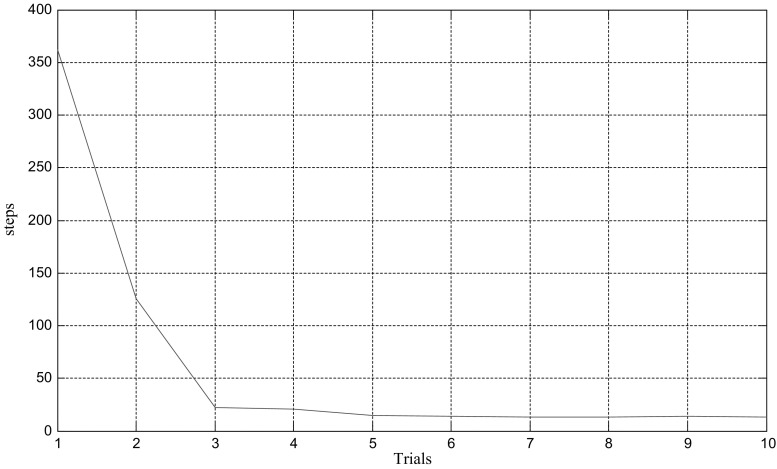
Statistics of path finding steps

As can be seen in Fig. 3, number of steps of mental exploration shows a declining trend overall. In this case, hundreds of steps are conducted to locate the target during the first random path-finding. Then after nine times of learning and optimization based on energy field gradient, a dozen of steps will suffice for mental exploration to find the target. The learning trend of path finding can be seen visually from statistical chart of step number (Fig. 4), where horizontal and vertical coordinates represent number of path findings and number of steps respectively.

**Fig. 5:**
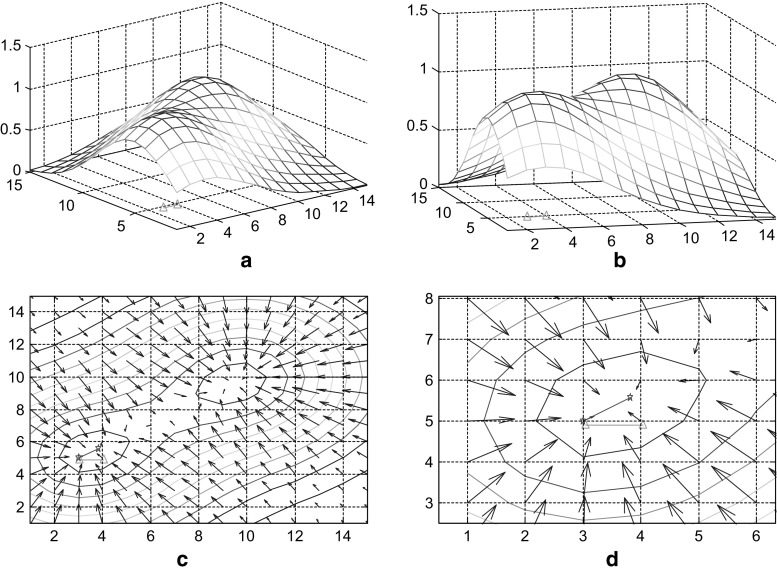
Energy field and navigation vector (6th step of 6th path finding)

Optimization procedure is guided by the energy field gradient as soon as the target is found by completely random path finding process. According to the model proposed in this paper, the form of the energy field is determined by target location, the topological structure of place cells with synaptic connection and synaptic strength. The energy field gradient is determined by these factors as well. As a result, any position explored by the system, will correspond to a specific energy field distribution, which will possess a certain gradient. This gradient, guiding the direction of next step of mental exploration, is the main part of the navigation vector (the other part is the random disturbance term). An example of the energy field and gradient navigation vector is presented in Fig. 5. This is the case before the 6th step in the 6th path-finding. Figure 5a, b depict the energy field before the 6th step of mental exploration from a different angle (rotated). Green line on the x–y plane represents the trajectory of step 6. Figure 5c, d are gradient vector maps of the upper energy field. Gradient at each point is depicted by the blue arrow (without normalization). The right diagram is the local amplification of the left one. The red line is the normalized gradient of current position of the system. This normalized gradient is the navigation vector for next step of exploration. The meaning of the green line is consistent with the upper diagrams. Due to the presence of random noise, there is a certain degree of difference between navigation vector and actual motion trajectory. In some cases, these two vectors may be more different, which results from a relatively stronger random vector.

**Fig. 6:**
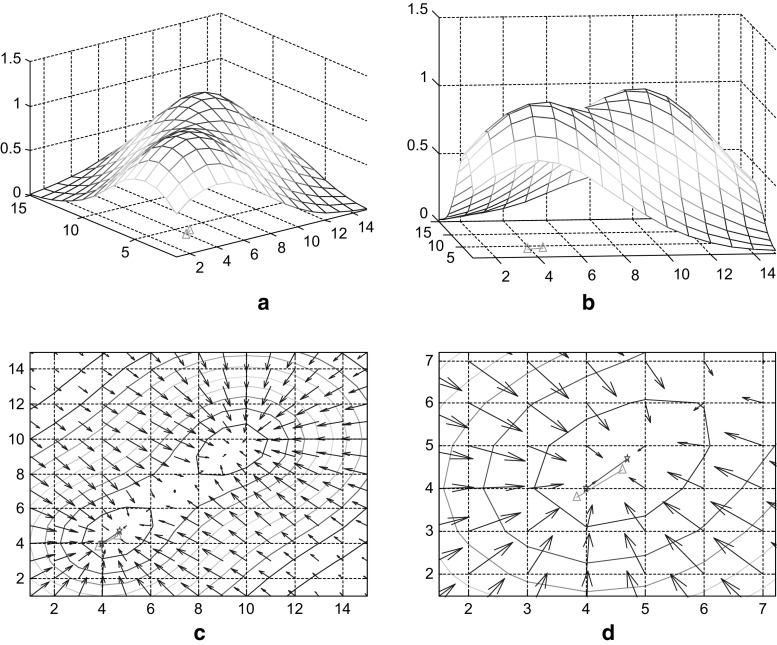
Energy field and navigation vector (6th step of 10th path finding)

Another example of 6th step in 10th path-finding is shown in Fig. 6. The meanings of all lines are same as in Fig. 5. There are hardly any differences between navigation vector and actual motion trajectory because, as mental exploration repeats, gradient navigation vector enhances and noise decays.

## Discussion

In traditional models, animals were required to undergo significant amounts of physical exploration to learn a new space, ranging from several minutes to many hours. Using mental exploration technique, a stationary simulation of animal exploration results in the formation of a representative virtual plane in the hippocampal CA3 region, thus simulating the process of real area exploration over the course of only a few seconds. Because of the rapid and responsive process involved, the mental exploration model may fulfill particular cognitive functions related to goal finding and path determination in practical settings (Zhu et al. 2013). On an abstract level, imagining or planning a path are all mental exploration. When circumstances permit its use, mental exploration can be faster, more energy-efficient, and safer than physical exploration for spatial problem-solving. Mental exploration can be separate from the original spatial knowledge generated by real exploration.

**Fig. 7:**
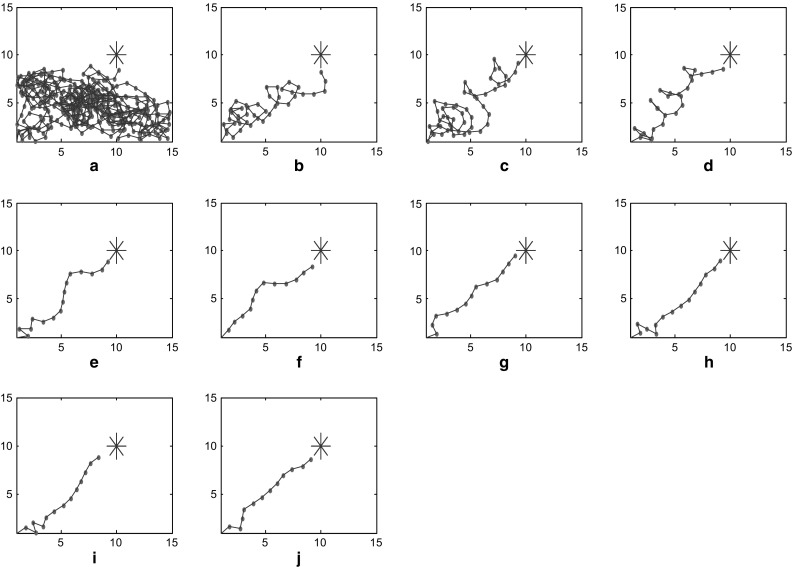
Trajectories of path finding process in small variance cases

**Fig. 8:**
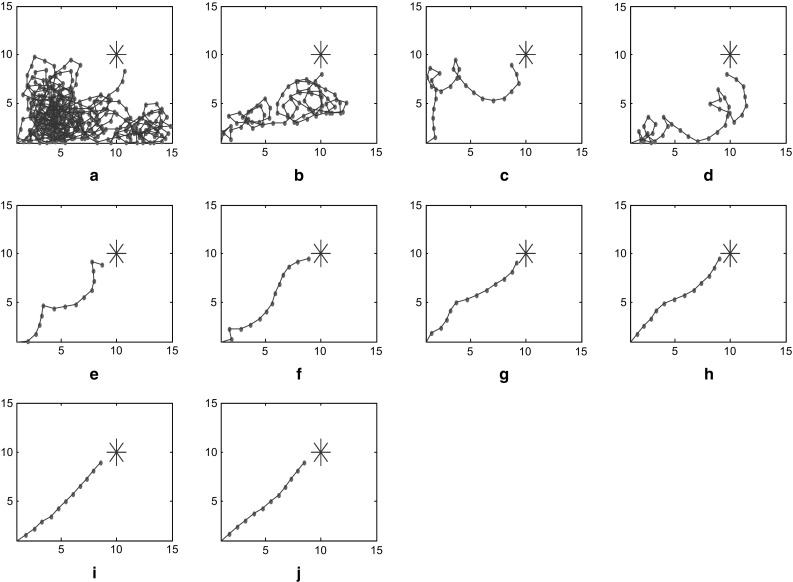
Trajectories of path finding process in large variance cases

**Fig. 9:**
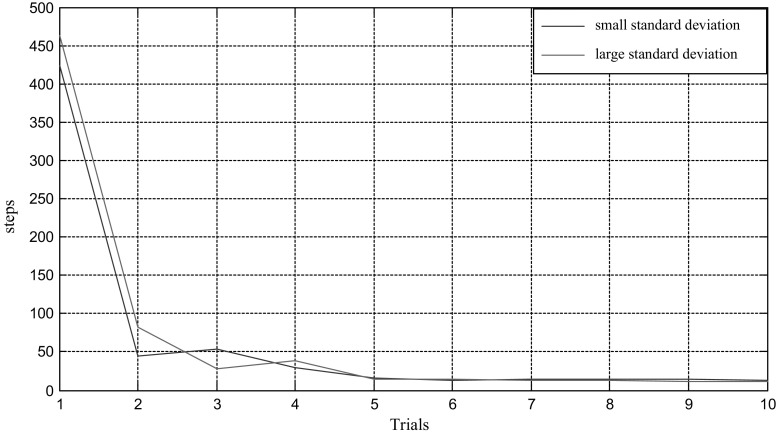
Statistics of path finding steps in two different cases

In this model, we proposed several factors that are crucial to the optimization procedure such as the increase of synaptic strength $\Delta \omega_{\text{ij}}$ and standard variance $\sigma_{1} ,\sigma_{2}$. $\Delta \omega_{\text{ij}}$ is determined by the learning rule in Eq. (16), while the differential is taken as a discrete form during the calculation. To detect the influence of parameter variations, we execute the model using different standard variances. These variances are set at $\sigma_{1} = \sigma_{2} = 3.52$ in one case and $\sigma_{1} = \sigma_{2} = 4.00$ in another. During the path finding process, the trajectories and steps are recorded in these two cases for comparison. Results are demonstrated as below in Figs. 7 and 8. These two figures show trajectory records of path finding process while standard variances are set to 3.52 and 4.00 respectively. There is no significant difference in the incipient few experiments between these two cases. However, the smaller-variance-case seems to have an optimization limit and is more likely to detour in the last few experiments. The larger-variance-case shows no such limit and tends to generate a better path. Statistics of path finding steps of these two cases in Fig. 9 demonstrate this result from another perspective. As we can see from Figs. 7i, j and 8i, j, the last few experimental results of the lager-variance-case are better than the smaller one. Fewer steps are taken in the final path of the larger variance case. If a certain part of the environment is searched many times, the synapse strength of place cells representing this area will be extremely large, since the Hebbian learning rule we adopted is not convergent. This will cause the path to concentrate in a single region and form a so-called “siege phenomenon”. This defect is probably the reason that the optimization of smaller-variance-case in the last few experiments is not so ideal. Fortunately, a relatively larger variance will solve this problem as is shown in Fig. 8. Larger variance means stronger synapse between target and current place cells and this property may balance the strong local synapse strength and results in generating more efficient navigation vectors. In addition, larger variance means that the attenuation of firing power caused by distance growth from place field center will slow down, which leads to more energy consumption. More efficient mental path means more energy cost; this seems quite reasonable for an animal. Of course, there is an alternative which is to improve the synaptic learning rule in this model. The synapse strength should be convergent as the experiment repeats, so the increase in synaptic strength $\Delta \omega_{\text{ij}}$ will be smaller in the later experiments, then the total synaptic change $\sum {\Delta \omega_{\text{ij}} }$ will convergent. This can be a topic for future studies.

**Fig. 10:**
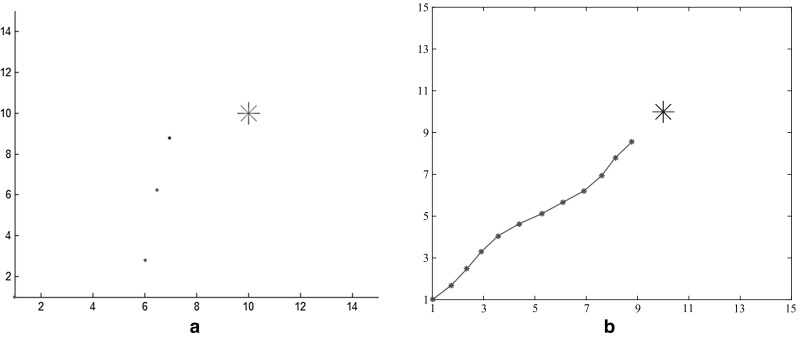
Comparative results of optimized paths

Comparing with former work (Zhu et al. 2013), the searching efficiency has been improved significantly in our model. The repeating trials are fewer and final path is shorter. Figure 10a is shows the fifteenth path in Zhu et al. (2013), which is an “S” shaped trajectory. Clearly it is not efficient enough. Figure 10b is the tenth path generated by our model. Unlike Fig. 10a, it is almost a straight line and repeating trials decrease 33 %.

## Conclusion

When the target to be explored is far away from the current position during the process of path-finding in spatial location field executed by rodent animal, the activity intensity of the place cell will gradually change with the distance. The farther distance leads to the lower activity intensity. In this work, based on such biology facts the power attenuation law of place cell is quantitatively described by Gauss distribution as widely existing in nature, and the spatial distributions of power of all place cells at the same time are regarded as an energy field. After constructing the energy field, we studied the feature of the gradient in this field. In combination with mental exploration proposed by Hopfield, energy field gradient is adopted for mental exploration, and the gradient acted as navigation vector, which greatly improves the efficiency of mental exploration. The results show that ten times of mental exploration lead to an optimal path. Compared with the published researches (Zhu et al. 2013), the method of neural energy field gradient may solve the path-finding problems and greatly improve the efficiency of mental exploration.

Our study, from the view of energy coding method, proves that synaptic plasticity plays an important role in spatial memory and mental exploration. And it also proves that adopting the global energy coding theory to mental exploration shows great advantages in path-finding problems. The results in our paper show that the synaptic connections between place cells have a decisive influence on the distribution of the energy field, so as to affect the gradient of the energy field gradient- navigation vector. However, the change of navigation vector affects the path-finding of mental exploration. Specifically, the repeat of the old path and the generation of the new path correspond to the certain firing patterns of place cells, which changes the synaptic connection strength in turn. Thus, energy field changes constantly under the impact of cycle feedback, so as to provide an optimal path for mental exploration through navigation. This means synaptic connection can serve as a bridge to build a bidirectional connection between energy field distribution and the path of mental exploration, one object can be studied to describe the other effectively. Then we analyzed the model we proposed and found an interesting property, which is that larger variance of the energy field will generate a better path. This finding may indicate a potential improvement for future studies. This work can also be regarded as the optimization of mental exploration concept with energy coding theory. While the original one is based on artificial neural network and has no biological meaning. Our results prove that it is effective to study path-finding problems with mental exploration based on energy coding theory, which has many potential applications in various research fields of artificial intelligence.
